# Cultural determinants of health for Aboriginal and Torres Strait Islander people – a narrative overview of reviews

**DOI:** 10.1186/s12939-021-01514-2

**Published:** 2021-08-12

**Authors:** Ebony Verbunt, Joanne Luke, Yin Paradies, Muriel Bamblett, Connie Salamone, Amanda Jones, Margaret Kelaher

**Affiliations:** 1 Centre for Health Policy, Melbourne School of Population and Global Health, The University of Melbourne, Melbourne, Australia; 2 Alfred Deakin Institute for Citizenship and Globalisation, Deakin University, Melbourne, Australia; 3 Victorian Aboriginal Child Care Agency, Melbourne, Australia

**Keywords:** Aboriginal and Torres Strait Islander, Indigenous, Cultural determinants of health

## Abstract

**Introduction:**

The cultural determinants of health centre an Indigenous definition of health, and have been linked to positive health and wellbeing outcomes. There is growing evidence for the importance of the cultural determinants of health; however, to date, no high-level overview of the evidence-base has been provided. Synthesising existing literature on cultural determinants of health for Aboriginal peoples in a single manuscript will highlight what we know, and what needs to be explored in future research. It will also contribute to global efforts to capture the evidence of cultural determinant approaches amongst Indigenous populations. We therefore endeavoured to identify cultural determinants and highlight their impact on Aboriginal health and wellbeing outcomes, and outline the relationship and interconnection of different cultural determinants of health.

**Methods:**

An overview of reviews was conducted. Medline (Ovid) and Scopus were searched using terms related to ‘cultural determinants of health’ and an ‘Aboriginal definition of health’. The database search was complemented by a web-based search of grey literature. Nine reviews were retrieved and included in our overview.

**Results:**

Family/community, Country and place, cultural identity and self-determination were strongly identified across reviews as having a positive impact on the health and wellbeing outcomes of Aboriginal peoples. Family/community and Country and place were found to be components of ‘culture’ that shaped cultural identity. Self-determination was outlined as a requirement for Aboriginal peoples to pursue their cultural, social, and economic rights.

**Discussion/conclusions:**

Cultural determinants are associated with health benefits for Indigenous peoples. A causal framework, developed to discuss the relationship and interconnection of the cultural determinants of health, demonstrates that cultural identity at an individual-level is important to benefiting from other cultural determinants of health. While self-determination and connection to culture and community-controlled organisations are integral factors to increase Aboriginal resilience and resistance and improve health and wellbeing outcomes. Further research is required to shift towards a multi-level understanding of the cultural determinants of health and to develop an Indigenous-led evidence-base around causal pathways. Such a shift would ensure priorities important to Indigenous peoples are captured in policy and practice.

**Supplementary Information:**

The online version contains supplementary material available at 10.1186/s12939-021-01514-2.

## Introduction

Research related to Indigenous peoples has traditionally taken a dominant social determinants of health approach, consequently positioning Indigenous identities in deficit relative to non-Indigenous populations and framing poor health as a consequence of an inability of Indigenous people to meet socioeconomic standards and ‘norms’ of dominant cultures [1–3]. This approach likely misses important structural, political, and cultural contexts, that present and enable opportunities to improve health. However, there is growing demand for researchers to centre Indigenous social realities and promote a strength-based perspective by focusing on the cultural determinants of health, as one social dimension of Indigenous peoples’ lives. Associate Professor Ray Lovett, an Aboriginal (Ngiyampaa/Wongaibon) epidemiologist, asserts that this approach will give ‘the ability of Aboriginal and Torres Strait Islander nations to inform and influence policy, program decisions and outcomes’ [4].

The cultural determinants of health centre an Indigenous definition of health, concentrating on ‘life-giving values from which individuals, families and communities can draw strength, resilience and empowerment’ [1, 3]. Similar to other Indigenous peoples, good health for Aboriginal peoples in Australia is a holistic concept. Health includes physical, social, emotional, spiritual, and ecological wellbeing, for the individual and the community, extending beyond a biomedical definition of health [5]. Culture can be defined as a way of life for people that is shared and learned [6] and is neither static nor confined to what is observable [7].

From an international perspective, there is consistent literature outlining the benefits of a cultural determinants approach. The effectiveness of a cultural determinants approach is said to have been demonstrated by the significant health gains achieved by the Indigenous people of Aotearoa New Zealand, during an era coined the ‘Maori renaissance’ [8]. Similarly, research in Canada found that suicide rates were distinctly lower for Indigenous communities with strong continuity of cultural practices compared to those where there had been dislocation of cultural practices [9]. The importance of cultural continuity for Indigenous health and wellbeing outcomes, is further outlined in a meta-synthesis of qualitative research in Canada and the United States [10]. Additionally, a recent review of international evidence supported the positive associations between wellbeing and the culture of Indigenous peoples; however, it noted that further mixed-method research was needed on the complex, causal pathways through which cultural determinants influence health and wellbeing outcomes [11].

In Australia, governments have increasingly turned to cultural determinants, with ‘connection to culture’ a key component of the *National Strategic Framework for Aboriginal People’s Mental Health and Social and Emotional Wellbeing 2017–2023* [12]. Cultural indicators are also a focus of the Australian Bureau of Statistics National Aboriginal and Torres Strait Islander Social Survey, with determinants relating to language, Country and identity collected [13].

Although dominant Australian institutions have only recently drawn their attention to the cultural determinants of health, the Aboriginal community-controlled sector have long focused on this strengths-based approach. For example, research conducted by the Victorian Aboriginal Health Service in the year 2000, revealed that community in Melbourne saw strong family (and extended family) links, friends, connection to community, connection to culture, sense of identity, aspirations, responsibility, and sports and creative activities as affirming cultural determinants of health [14]. More recently, a number of tools have been developed to capture cultural determinants and measure their effect on health [15–17]. One such example is the Aboriginal Resilience and Recovery Questionnaire, which examines psychological factors related to community, cultural, relational and individual, and their association with resilience and recovery from trauma [18]. Further, a longitudinal study, Mayi Kuwayu, is currently being conducted to assess how determinants such as connection to Country, cultural practices, spirituality, and language use is linked to Aboriginal wellbeing [19].

There is growing evidence for the importance of the cultural determinants of health for Indigenous peoples; however, to date, no high-level overview of this evidence has been conducted. By drawing on high-grade evidence [20], our overview will provide a more comprehensive analysis of the cultural determinants of health than that of existing research available; highlighting both what we know, and what needs to be explored in future research. Additionally, by outlining our current understanding of what cultural determinants of health are valued in the literature, and how they interact to improve health and wellbeing outcomes, this review contributes to global efforts to capture the evidence of cultural determinant approaches amongst Indigenous populations. With a growing body of research, the role that cultural determinant of health approaches have in contributing to more equitable outcomes for Indigenous peoples can be realised. We therefore endeavoured to provide a broad overview of cultural determinants and health and wellbeing outcomes for Aboriginal peoples, and outline the relationship and interconnection of different cultural determinants of health. We then draw on the available evidence to discuss a potential cultural determinants of health framework.

## Method

A narrative overview of reviews was conducted, rather than a systematic review or meta-analysis, neither of which was possible due to the heterogeneity of included reviews. The approach taken to the search and inclusion of reviews was systematic.

### Data sources and searches

Two databases were searched from January 2000 to February 2019: Medline (Ovid) and Scopus. The search strategy was generated with the assistance of a research librarian, with terms including previously identified cultural determinants of health and an Aboriginal definition of health. A full list of search terms by database is provided in Additional file 1. The database search was complemented by a web-based search of grey literature. Searches used the Google search engine and included reputable Aboriginal health websites, such as the Australian Institute of Aboriginal and Torres Strait Islander studies, Australian Indigenous HealthInfoNet, and the Cooperative Research Centre for Aboriginal and Torres Strait Islander Health.

### Inclusion and exclusion criteria

Articles were included if they met the following criteria: (i) publications = reviews; (ii) cultural determinant(s) of health discussed; and (iii) available in full-text. Articles were excluded if they met the following criteria: (i) empirical studies; (ii) reviews focused on the cultural competency/safety of health care; (iii) reviews without disaggregated information on the cultural determinants of Aboriginal Australians; and (iv) reviews in languages other than English.

### Screening of records and data extraction

The searches from each database were imported into Endnote X8 and duplicates were excluded. One author (EV) screened titles and abstracts for relevance and full-text studies for eligibility. Two authors (EV and JL) extracted the following information from the reviews into a predesigned form: author(s); year of publication; number of included studies; aim; cultural determinant(s) discussed; effect on health and wellbeing; conclusions; and recommendations for future research.

## Results

### Search results

**Fig. 1:**
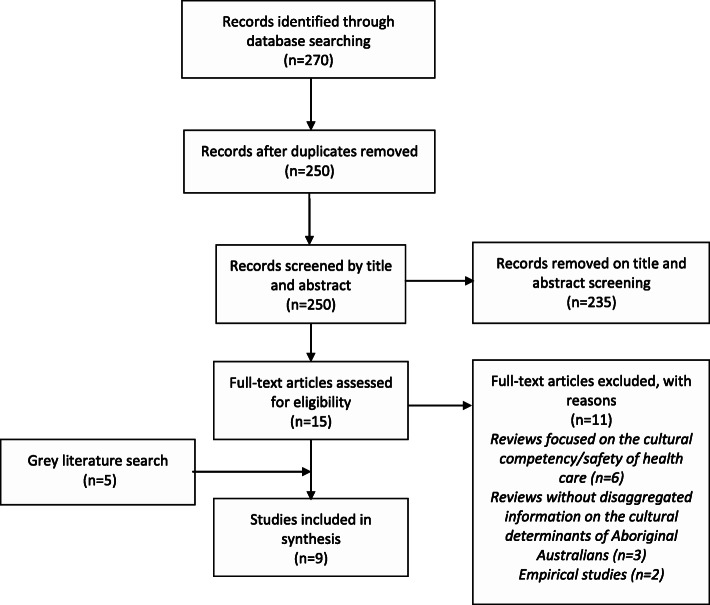
Flow diagram for retrieval of reviews

A total of 270 studies were identified during database searches and imported in Endnote X8, with 20 duplicate studies removed before screening (Fig. 1). Following title and abstract screening, we excluded 235 studies, leaving 15 studies for full-text screening. Following full-text screening, we excluded 11 studies, of which: 6 were reviews focused on the cultural competency/safety of health care, 3 were reviews without disaggregated information on the cultural determinants of Aboriginal Australians, and 2 were empirical studies. The grey literature search identified a further five reviews. In total, nine reviews were included in our overview of reviews.

### Characteristics of reviews

Nine reviews were published between 2005 and 2018 – with four published during or before 2010, and the remaining five published after 2010. Five were literature reviews [21–25], two were systematic reviews [26, 27], one was a scoping paper [28] and one was a meta-synthesis of qualitative studies [29].

All nine reviews included primary research studies conducted in Australia and which targeted only Aboriginal peoples. Two reviews [21, 22] focused on studies that occurred in remote Australia, with the remaining seven reviews including studies in various types of environments (urban, rural, remote). Three reviews [21, 22, 25] were focused on understanding the connection to Country and health, one review [28] was on the link between self-determination and health, one focused on Aboriginal peoples’ understanding of mental health [29], one focused on Aboriginal peoples understanding of physical health [26], and three reviews [23, 24, 27] were focused on cultural elements important to Aboriginal health.

### Overview of cultural determinants and health and wellbeing outcomes

While cultural determinants often intersected, there were four determinants that were strongly identified across multiple reviews included in the overview – family/community, Country and place, cultural identity, and self-determination. Table 1 highlights how these cultural determinants can be understood to have an impact on health and wellbeing outcomes.

**Table 1:** Cultural determinants and impact on the health and wellbeing of Aboriginal peoples

First Author [reference number] Year	**Family/community**	**Country and place**	**Cultural identity**	**Self-determination**
Berry [21] 2010	Unknown yet whether community-led caring-for-Country projects could produce both benefits for country and personal empowerment	Greater physical activity (associated with caring-for-country projects) is linked to better mental health directly and because it improves physical health, itself strongly linked to mental health [30, 31]	Caring for Country could support connectedness to identity and generate powerful co-benefits for social and emotional wellbeing	To reduce the current preventable burden of disease, we must address lack of control as one aspect of a broader experience of Aboriginal powerlessness [5, 32]
Burgess [22] 2005	Cohesion with kin, ancestors and geography are all important factors in the formation of collective esteem and efficacy [33, 34]	Engagement with Country provides opportunities for physical activity, improved diet as well as boosting individual autonomy and self-esteem [35, 36]	“Our identity as human beings remains tied to our land… Destroy this relationship and you damage – sometimes irrevocably – individual human beings and their health” [37]	Renewed ties to Country can form the basis of reinvigorated governance and can develop a sense of autonomy and mastery over life [38, 39]
Salmon [23] 2018	Social support, in the form of community affection, is a strong determinant of health for Aboriginal women [40]	Caring for country has benefits for the socio-political, cultural, economic, and physical and emotional wellbeing of Aboriginal peoples [22, 41–45]	Culture is critically important – it is the central core of Aboriginal child health and wellbeing	Leadership is critical to the long-term survival and growth of Koori [Aboriginal people from New South Wales and Victoria] communities [46]
VicHealth [24] 2011	Family and kinship networks and community connections can promote health and can be harmful to health [47]	Programs that involve Aboriginal Natural and Cultural Resource Management are associated with lower levels of risk factors for CVD and diabetes and improved self-esteem [48]	Caring for Country appears to be linked to improved identity and is recommended as a means to improving health [49]	A requirement for reversing colonisation is self-determination. Self-determination helps people restore control over their lives and destinies
Weir [25] 2011	Not reported	Caring for Country has benefits for individual health and wellbeing, and the health of communities	A relationship between identity, autonomy and wellbeing and caring for Country [48]	Control over life is an important determinant of wellbeing [50]
Dahlberg [26] 2018	Family determines a child’s level of activity and type of sport they practice [51, 52]	A big emphasis is placed on physical and social environment when it comes to physical activity	Evidence of the positive impact in the strong sense of collective identity and pride that is experienced when part of a team [51]	Not reported
MacLean [27] 2017	Welcoming extended family members to attend antenatal care and childbirth supports the expression of cultural identity and can decrease perinatal morbidity [53]	Activities on Country can be used to improve health [54]	Programs that include components to enable and support Aboriginal peoples to express cultural identity can have positive health and wellbeing effects	Not reported
Behrendt [28] 2017	Not reported	It is not possible to conceive of Aboriginal health determinants without appreciating the inseparable connection between wellbeing and Country	Valuing Aboriginal knowledge and cultural beliefs and practices is necessary for positive cultural identity and social and emotional wellbeing [55]	A link between self-determination and improved health and wellbeing; however, demonstrating this link can be challenging [55]
Ypinazar [29] 2007	Family is considered pivotal to emotional, physical and cultural health [56]	Not reported	A positive sense of identity is connected to individual and community wellbeing [56, 57]	Not reported

### Relationship and interconnection of cultural determinants of health

As shown in Table 1, the determinants of family/community and Country and place often intertwine and are mutually reinforcing in their effect on Aboriginal health and wellbeing outcomes. These determinants were often expanded in reviews to include other elements, such as – connection across generations, and participation in community activities and events, sports, and arts-based cultural expression. We therefore chose to group these determinants together under the term ‘culture’, below.

#### Culture

Maclean et al. [27] outlined how connection to Country does not necessarily have to be physical, describing how mapping one’s ‘travel’ through Country can contribute to positive health outcomes [58]. Similarly, Salmon et al. [23] found that connection to Country in urban areas can be maintained by teaching children about the ‘geographical boundaries of their Country and significant places or plants for medicine and tools and through telling stories about experiences of previous generations’ [59]. Further, Burgess et al. [22] outlined that engagement with Country can provide a significant source of social cohesion for Aboriginal peoples, as it often involves group activity. It was also highlighted in a review by Dahlberg et al. [26] that in urban areas, connection to family and community can occur through participation in sporting teams [51].

Connection across generations was an identified determinant, with Salmon et al. [23] outlining how Elders as ‘custodians of traditional knowledge, history, culture and language’ can play a vital role in the transfer of knowledge to younger generations. Based on research on the keys to healing for Aboriginal children and young people, and securing their social and emotional wellbeing [60], it was concluded that the transfer of knowledge through Elders provided protective factors for the health and wellbeing of the next generation. Additionally, VicHealth [24] outlined how a key component of Aboriginal child health and wellbeing in urban areas included respect for Elders [61]. Salmon et al. [23] reported that local leadership should also include youth leaders, who as drivers of social change, need to be developed and supported [43, 62–65].

Arts and crafts, music, dance, theatre and writing or telling stories are elements of arts-based cultural expression that appear important to the wellbeing of Aboriginal peoples. Salmon et al. [23] proposed that they are an important means of passing on spiritual awareness, with research finding that art, song and ceremony ‘result in resilience in the face of racism and generational trauma as a result of colonisation and resistance to the colonising of Aboriginal spirituality’, with reclaiming spirituality a means to reclaiming identity [66].

#### Cultural identity

Culture (family/community, country and place, connection across generations, and participation in community activities and events, sports and arts-based cultural expression) are outlined in the included reviews as integral components of cultural identity, with Weir, Stacey and Youngtob [25] stating that ‘by affirming relationships with Country, one is also affirming deep-seated dimensions of one’s cultural identity’. Cultural identity was described by Salmon et al. [23] as also being shaped by ‘respect for Elders, gender and age roles, language, art and ceremony…’. Similarly, Ypinazar et al. [29] outlined the importance of ‘storytelling, ceremonies, ancestors, sacred sites, and tribal areas’ for positive cultural identity and Aboriginal wellbeing [56, 57, 67].

#### Self-determination

The review by VicHealth [24] outlined self-determination as a requirement for decolonisation. Self-determination allows Aboriginal peoples to pursue their cultural, social, and economic rights, with Berry et al. [21] concluding that to ‘reduce preventable burden of disease, lack of control and powerlessness must be addressed.’ Self-determination requires Aboriginal peoples to be involved ‘in every layer of decision-making’ [28] and as highlighted by Salmon et al. [23], this means leading the discourse around the concept of self-determination itself.

The importance of community control was exemplified by Behrend, Jorgensen and Vivian [28] who concluded that Community Controlled Organisations contribute to many positive outcomes for Aboriginal peoples, due to their ‘flexibility’ and ability to ‘build partnerships within and between communities and within government departments and NGOs’ [68].

## Discussion

Evidence presented in our overview of reviews supports existing national and international research that the cultural determinants of health can positively impact the health of Indigenous populations [8–11, 14]. Family/community, Country and place, cultural identity and self-determination were strongly identified across reviews as having a positive impact on the health and wellbeing outcomes of Aboriginal peoples. The determinants of family/community and Country and place were often associated with other elements, such as – connection across generations, and participation in community activities and events, sport, and arts-based cultural expression, with these determinants subsequently grouped under the term ‘culture’ [21–29].

**Fig. 2:**
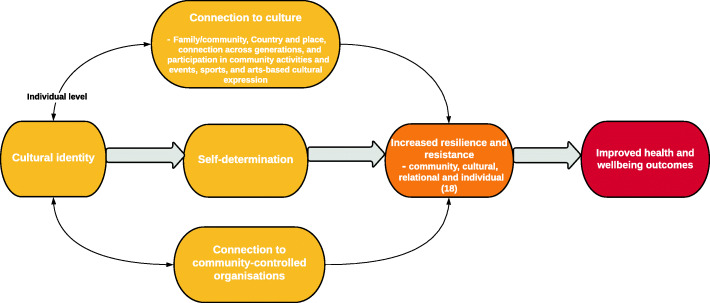
A causal framework of the cultural determinants of health for Aboriginal peoples

To discuss the relationship and interconnection of the cultural determinants of health outlined in the findings, we have developed a causal framework (Fig. 2). Factors of the Aboriginal Resilience and Recovery Questionnaire have been included to demonstrate how the effect of cultural determinants on health and wellbeing could be measured [18]. As outlined in Fig. 2, cultural identity at an individual level is important to benefiting from other cultural determinants of health. While self-determination and connection to culture and community-controlled organisations, are integral factors to positive health and wellbeing outcomes. Connection to culture (family and community, Country and place, connection across generations, and participation in community activities and events, sports, and arts-based cultural expression) are mutually reinforcing, shaping cultural identity, and improving Aboriginal health and wellbeing outcomes. Although cultural identity on an individual level is necessary for improving the health of Aboriginal peoples, self-determination at a broader level is a requirement for Aboriginal peoples to pursue their cultural, social, and economic rights. Connection to culture and community-controlled organisations are both seen as contributing to resilience and resistance as measured by the Aboriginal Resilience and Recovery Questionnaire [18], and in turn, improving the health and wellbeing outcomes of Aboriginal peoples [21–29].

Whilst our overview of reviews has highlighted our current understanding of what cultural determinants are valued in the literature, and how they interact to improve health and wellbeing outcomes, it has also highlighted gaps in the current evidence-base – providing direction for future research.

Although culture is not static [7], many studies conceptualised it to be so, with a primary focus on the influence of cultural determinants at an individual-level. Failure to consider the broader impact on families and communities has most likely led to an underestimation of the benefits associated with exposure to culture, and the value to be gained by investing in systemic and structural changes required to strengthen culture and health now and into the future. While in saying this, we are also cognisant that the literature on the cultural determinants of health has tended to focus on individuals and their participation in culture, without consideration to the systemic and structural factors that facilitate and impinge on participation, and themselves shape culture. In considering the benefits of a cultural determinants of health approach, recent work in this space highlights a need to look beyond culture and the community; to social, economic, and political dimensions in which communities are embedded, and how they in-turn interact with cultural determinants [69].

There are also limitations to our own study that should be considered, particularly when utilising our causal framework of the cultural determinants of health for Aboriginal peoples. We summarise cultural determinants of health at a national level, when we know there is no homogenised national Aboriginal culture in Australia. We also provide a broad overview of the determinants and their associations, and as such, we recognise the determinants and pathways described in the literature and included in Fig. 2, are unlikely to capture culture in its full complexity. Although studies within reviews included a diversity of Aboriginal peoples, a more nuanced understanding of the cultural determinants of health is required, and should come from the language, social or nation groups of Indigenous peoples themselves.

Lastly, we did not assess the extent to which Aboriginal people were involved in studies included reviews are based upon, with the potential that the knowledge and representation of Aboriginal culture is not located in the lived experience of Aboriginal peoples.

Future research is warranted to shift towards a multi-level understanding of the cultural determinants of health, and to develop an Indigenous-led evidence-base around causal pathways. Such a shift would ensure research is respectful and credible, and that priorities important to Indigenous peoples are captured in policy and practice.

## Conclusions

By drawing on high-grade evidence [20], our overview of reviews contributes to global efforts to capture the evidence of cultural determinants approaches amongst Indigenous populations. Our causal framework demonstrates that cultural identity at an individual-level is important to benefiting from other cultural determinants of health. While self-determination and connection to culture and community-controlled organisations are integral factors to increase Aboriginal resilience and resistance and improve health and wellbeing outcomes. We have also highlighted gaps in the current evidence-base, with further research required to shift towards a multi-level understanding of the cultural determinants of health and to develop an Indigenous-led evidence-base around causal pathways.

## Supplementary Information

**Additional file 1. ** Search strategy.

## Data Availability

All data generated or analysed during this study is included in this published article.
