# Supplementary material for: Cultural determinants of health for Aboriginal and Torres Strait Islander people – a narrative overview of reviews
Source: Int J Equity Health. 2021 Aug 12;20:181. doi: 10.1186/s12939-021-01514-2 (PMC8359545; doi:10.1186/s12939-021-01514-2)
Supplement: Supplementary file 1 — Additional file 1. Search strategy. [file 12939_2021_1514_MOESM1_ESM.pdf]

Additional file 1: Search strategy

Scopus: (Aboriginal or 'Torres Strait Islander*'or Indigenous) AND (Australia) AND (social wellbeing or emotional wellbeing or health or wellbeing or safety) AND (cultur* or spiritual or identity or family or community or country or land or language or self-determination or empowerment).

Medline (Ovid):

1. Oceanic Ancestry Group/
2. Indigenous
3. Aborigin*
4. Torres Strait
5. or/1-4
6. exp Australia/
7. (Australia* or Northern Territory or Queensland or New South Wales or Victoria or Tasmania)
8. 6 or 7
9. 5 and 8
10. (social wellbeing or emotional wellbeing or health or wellbeing or safety)
11. (cultur* or spiritual or identity or family or community or country or land or language or self-determination or empowerment)
12. 9 and 10 and 11
